# Protocol for non-viral HDR-based CRISPR/Cas9 platform for small custom editing in primary T cells

**DOI:** 10.3389/fgeed.2026.1817322

**Published:** 2026-09-30

**Authors:** Katariina Mamia, Anniken Solveig Matheson Sollano, Vanja Lundberg, Svetlana Vakkilainen, Eliisa Kekäläinen, Camilla Schalin-Jäntti, Eira Leinonen, Børre Fevang, Juha Grönholm, Timi Martelius, Shiva Dahal-Koirala, Emma Maria Haapaniemi

**Affiliations:** 1 Norwegian Centre for Molecular Biosciences and Medicine, University of Oslo, Oslo, Norway; 2 Department of Pediatrics, Oslo University Hospital, Oslo, Norway; 3 Precision Immunotherapy Alliance, University of Oslo, Oslo, Norway; 4 School of Biosciences and Medicine, University of Surrey, Guildford, Surrey, United Kingdom; 5 Department of Pediatrics, Institute of Clinical Sciences, the Sahlgrenska Academy at the University of Gothenburg, Gothenburg, Sweden; 6 Department of Rheumatology and Inflammation Research, Institute of Medicine, the Sahlgrenska Academy at the University of Gothenburg, Gothenburg, Sweden; 7 Children’s Hospital and Pediatric Research Center, University of Helsinki and Helsinki University Hospital, Helsinki, Finland; 8 Clinical Microbiology, HUS Diagnostic Center, Helsinki University Hospital and University of Helsinki, Helsinki, Finland; 9 Translational Immunology Research Program, University of Helsinki, Helsinki, Finland; 10 Institute for Molecular Medicine Finland and Helsinki Institute of Life Science, University of Helsinki, Helsinki, Finland; 11 Endocrinology, Abdominal Center, Helsinki University Hospital, Helsinki, Finland; 12 University of Helsinki, ENDO-ERN (European Reference Network on Rare Endocrine Conditions), Helsinki, Finland; 13 Folkhälsan Institute of Genetics, and Stem Cells and Metabolism Research Program, University of Helsinki, Helsinki, Finland; 14 Institute of Clinical Medicine, University of Oslo, Oslo, Norway; 15 Section of Clinical Immunology and Infectious Diseases, Oslo University Hospital, Rikshospitalet, Oslo, Norway; 16 Pediatric Research Center, New Children’s Hospital, University of Helsinki and HUS Helsinki University Hospital, Helsinki, Finland; 17 Division of Hematology, Oncology, and Stem Cell Transplantation, New Children’s Hospital, HUS Helsinki University Hospital, Helsinki, Finland; 18 Inflammation Center, Department of Infectious Disease, Helsinki University Hospital and University of Helsinki, Helsinki, Finland

**Keywords:** CRISPR/Cas9, genome editing, homology directed repair (HDR), inborn errors of immunity (IEI), T cells

## Abstract

CRISPR/Cas9 enables precision gene editing via homology-directed repair (HDR) for mutation correction and disease modelling. Here we present a customizable T cell single nucleotide variant (SNV) correction platform based on non-viral HDR, previously described in Mamia et al. *Precision T Cell Correction Platform for Inborn Errors of Immunity*. Molecular Therapy, 2025. To enable reproducible application, we provide a comprehensive, step-by-step 8-day HDR workflow for editing primary T cells, covering CRISPR/Cas9 reagent design, custom genomic editing from start to finish, optimization and validation strategies, on-target detection, and flow cytometric assessment of T cell phenotype and exhaustion. The platform separates locus-specific reagent optimization from a fixed cellular workflow, enabling implementation of new genomic targets without modifying the downstream T cell editing pipeline. Using this workflow, we have achieved up to 80% editing efficiency across multiple loci and donors. We have further optimized the T cell culture platform for PBMC stimulation to promote CD4^+^ and CD8^+^ T cell activation and proliferation. We validated this culture platform in 33 patients with Inborn Errors of Immunity using flow cytometry and observed that the defined culture conditions maintain low T cell exhaustion and support persistence of memory T cell populations. The protocol was developed under research-grade conditions but supports scalability and transition to preclinical and clinical GMP workflows. Overall, this adaptable platform is a broadly applicable framework for precision T cell engineering across diverse genomic targets offering opportunities for both *in vitro* disease modeling and therapeutic genome correction.

## 1 Introduction

CRISPR/Cas9 gene editing is a promising tool to correct pathogenic variants for autologous T cell therapies, targeting monogenic diseases such as Inborn Errors of Immunity (IEI). Furthermore, it can be used as a tool for disease modelling to study normal and pathological variations of the immune system (Mamia et al., 2025).

Here we present a detailed protocol for an efficient and customizable T cell single nucleotide variant (SNV) correction platform based on homology-directed repair (HDR). We use a modular design in which locus-specific reagents are optimized independently of the core T cell culture and editing platform. The protocol details every step of the process, which starts with custom CRISPR/Cas9 reagent design of guide-RNAs (gRNAs) and repair templates for editing a novel target with no previously published reagents. Furthermore, we describe the strategy of reagent design to assess on-target HDR editing using droplet digital PCR (ddPCR). Next, we present the workflow of gene editing T cells using nucleofection and CRISPR ribonucleoprotein (RNP) complexes for efficient editing that preserves high cell viabilities and up to 80% HDR (Mamia et al., 2025). Furthermore, the cells generated using this platform can be analyzed using a wide range of user-defined techniques to interrogate DNA, RNA, protein, and functional outcomes (Mamia et al., 2025). We have further optimized the T cell culture platform for PBMC stimulation to promote CD4^+^ and CD8^+^ T cell activation and proliferation. This approach has been validated in 33 patients with IEI using a flow cytometry panel to assess immune cell composition, memory phenotype, and exhaustion status at the end of the workflow. The defined culture conditions maintain low T cell exhaustion and support the persistence of memory T cell populations.

This T cell gene editing workflow (Figure 1) provides a broadly transferable platform for precision editing across diverse gene targets, supporting both translational gene correction and experimental disease modelling.

**FIGURE 1:**
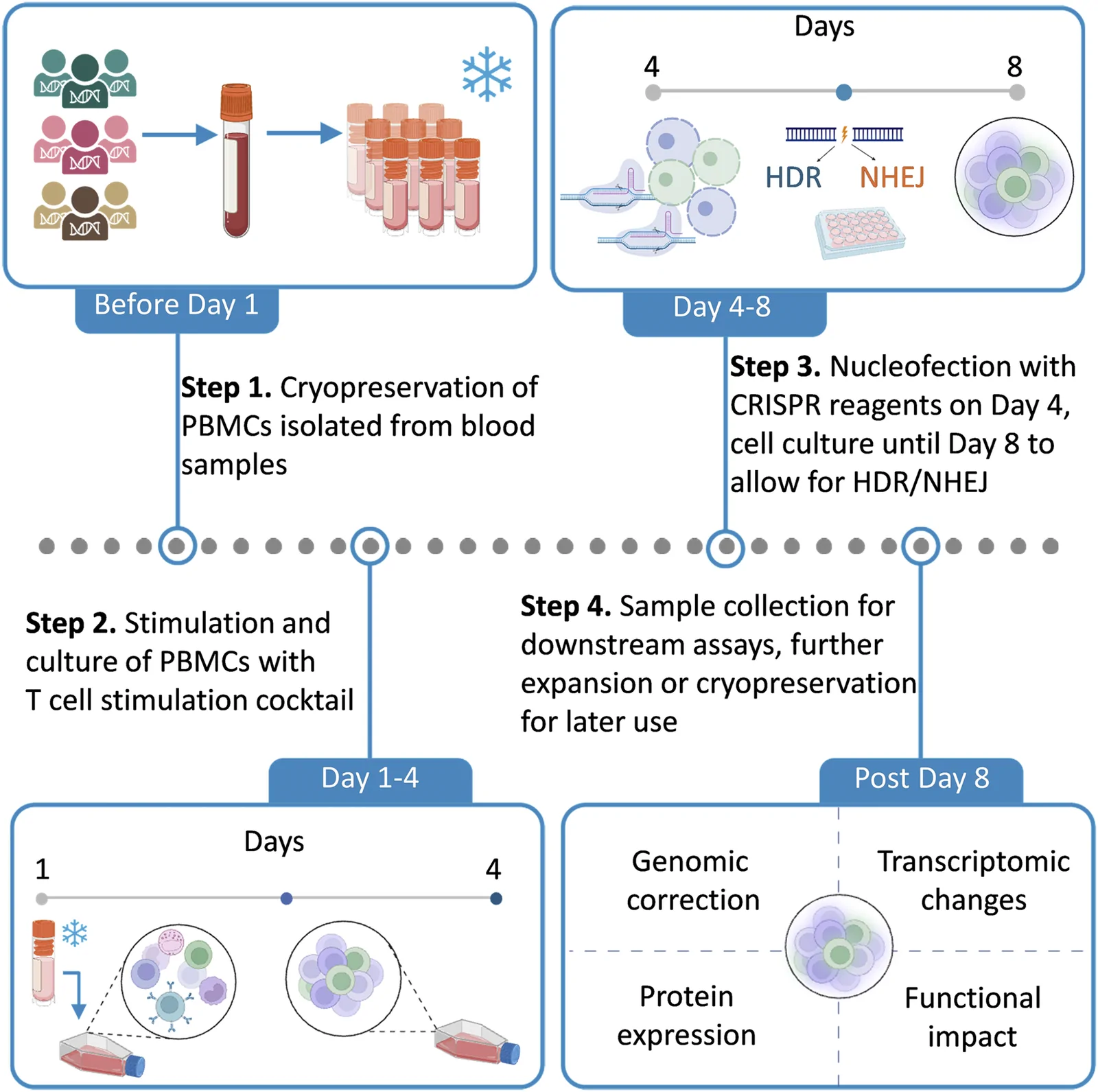
Eight-day workflow for CRISPR/Cas9-mediated HDR-based precision editing in primary T cells. Schematic overview of the stepwise protocol for CRISPR/Cas9-mediated homology-directed repair (HDR) editing in patient-derived and healthy donor primary T cells. Peripheral blood mononuclear cells (PBMCs) are isolated from whole blood and cryopreserved. PBMCs are then thawed, stimulated, and cultured using a T cell stimulation cocktail to activate and expand T cells prior to genome editing. The cells are nucleofected with CRISPR/Cas9 reagents, followed by culture until Day 8 to allow DNA repair through homology-directed repair (HDR) or non-homologous end joining (NHEJ). Finally, the cells are harvested for downstream analyses, further expansion, or cryopreservation. Downstream applications include assessment of genomic correction efficiency (e.g., droplet digital PCR, amplicon sequencing), protein expression (e.g., flow cytometry, mass spectrometry), transcriptomic changes (bulk or single-cell RNA sequencing), and functional impact (e.g., proliferation assays), providing a comprehensive platform for evaluating precision genome editing outcomes.

### 1.1 Materials and equipments

**Table:** 

Reagent or resource	Source	Identifier
Lymphoprep	Stemcell Technologies	#18061
LeukoSep tube (50 mL)	Greiner	#227288
PBS (pH 7.4)	Thermo Fisher Scientific	#10010023
40 µm Cell Strainer	Corning	#352340
CryoStor freezing medium	Stemcell Technologies	#100-1061
ImmunoCult™-XF T Cell Expansion Medium	Stemcell Technologies	#10981
ImmunoCult™ Human CD3/CD28 T Cell Activator	Stemcell Technologies	#10971
ImmunoCult™ Human CD3/CD28/CD2 T Cell Activator	Stemcell Technologies	#10970
Recombinant human IL-2	Miltenyi Biotec	#130-097-742
Recombinant human IL-7	Miltenyi Biotec	#130-093-937
Recombinant human IL-15	Miltenyi Biotec	#130-093-955
Alt-R S.p. Cas9 Nuclease V3	Integrated DNA Technologies	#1081058
Armadillo PCR plate (96-well)	Thermo Fisher Scientific	#AB3396
Microplate Aluminum Sealing Tape	Corning	#6570
TheraPEAK™ P3 solution set	Lonza	#G4LP3-22500
DNeasy Blood & Tissue Kit	Qiagen	#69506
Qubit™ dsDNA HS Assay Kit	Thermo Fisher Scientific	#Q32854
ddPCR plates, 96 well, semi-skirted	Bio-Rad	#12001925
ddPCR Supermix for Probes (No dUTP)	Bio-Rad	#1863025
Piercable Foil Heat Seal	Bio-Rad	#1814040
Automated Droplet Generator	Bio-Rad	N/A
PX1 PCR Plate Sealer	Bio-Rad	N/A
QX200™ Droplet Reader	Bio-Rad	N/A
QX Manager	Bio-Rad	N/A
96-well V bottom plate	Thermo Fisher Scientific	#249935
CoolCell container	Corning	N/A
15 mL Falcon tube	Corning	#352096
50 mL Falcon tube	Corning	#352070
SafeSeal reaction tube, 1.5 mL	Sarstedt	#72.706.006
Lonza 4D-Nucleofector	Lonza	N/A
Lonza 16-well strip or 96-well nucleofection plate	Lonza	N/A
TheraPEAK™ P3 solution set	Lonza	#G4LP3-22500
AbC™ Total Antibody Compensation Bead Kit	Invitrogen	#A10513
LIVE/DEAD™ Fixable Violet Dead Cell Stain Kit, for 405 nm excitation	Thermo Fisher	#L34963
Alexa Fluor® 488 anti-human CD3 Antibody	Biolegend	#300415
PerCP/Cyanine5.5 anti-human CD62L Antibody	Biolegend	#304824
Pacific Blue™ anti-human CD19 Antibody	Biolegend	#302232
Pacific Blue™ anti-human CD14 Antibody	Biolegend	#301828
Pacific Blue™ anti-human CD56 (NCAM) Antibody	Biolegend	#304629
Brilliant Violet 570™ anti-human CD8a Antibody	Biolegend	#301037
PE/Cyanine7 anti-human CD45RA Antibody	Biolegend	#304125
BD Horizon™ V450 Mouse Anti-Human CD11c	BD Biosciences	#560369
BD Horizon™ PE-CF594 Mouse Anti-Human CD279 (PD-1)	BD Biosciences	#565024
BD Pharmingen™ Alexa Fluor® 647 Mouse Anti-Human TIM-3 (CD366)	BD Biosciences	#565559
BD™ APC-H7 Mouse Anti-Human CD4	BD Biosciences	#641398
FcR Blocking Reagent human	Miltenyi Biotec	#130-059-901
eBioscience Flow Cytometry Staining Buffer	Invitrogen	#00-4222-26
5 mL Round Bottom Polystyrene Test Tube	Corning	#352054
LSR II Flow Cytometer	BD Biosciences	N/A

### 1.2 IEI patiens used in the study

**Table:** 

Patient number	Affected gene	Disease	Mode of inheritance
1	STAT1	STAT1 gain of function disease	AD
2	STAT1	STAT1 gain of function disease	AD
3	STAT1	STAT1 gain of function disease	AD
4	STAT1	STAT1 gain of function disease	AD
5	STAT3	STAT3 gain of function disease	AD
6	CTLA4	CTLA4 haploinsufficiency	AD
7	AIRE	Autoimmune polyendocrinopathy-candidiasis-ectodermal dystrophy	AR
8	RMRP	Cartilage hair hypoplasia	AR
9	RMRP	Cartilage hair hypoplasia	AR
10	BTK	X-linked agammaglobulinemia	XLR
11	CYBB	X-linked chronic granulomatous disease	XLR
12	WAS	Wiskott-aldrich syndrome	XLR
13	ADA2	Deficiency of ADA2 (DADA2)	AR
14	LIG1	LIG1 deficiency/unknown	Unknown
15	STAT3	STAT3 dominant negative	AD
16	IL2RG	Atypical SCID/unknown	XLR
17	SASH3	SASH3 deletion/unknown	XLR/unknown
18	CARMIL2	CARMIL2 deficiency	AR
19	STAT3	STAT3 gain of function	AD
20	IKZF2	Immunodeficiencies affecting cellular and humoral immunity	AD/unknown
21	CARMIL2	CARMIL2 deficiency	AR
22	TOM1	TOM1 deficiency	Unknown
23	HYOU1	HYOU1 loss of function	Unknown
24	PAK2	Unknown	AD
25	BTK	X-linked agammaglobulinemia	XLR
26	STAT3	STAT3 dominant negative	AD
27	RMRP	Cartilage hair hypoplasia	AR
28	BACH2	BACH2 deficiency	AD
29	PSMB10	PSMB10 (SCID phenotype)	AD
30	PSMB10	PSMB10/unknown	No disease causing variant
31	PIK3CD/PIK3R1	Activated PI3K delta syndrome	AD
32	CTLA4	CTLA4 haploinsufficiency	AD
33	JAK1	JAK1 GOF	AD

## 2 Methods

### 2.1 Target sequence identification and visualization

This step outlines the initial phases of precision editing a novel locus, where either a wild-type sequence is used to design editing tools for healthy controls, or a patient-specific sequence is used for mutation correction or another type of edit. For a novel target that does not have previously published CRISPR/Cas9 reagents, the protocol starts here. If previously published CRISPR/Cas9 reagents are available and preferred, proceed to Step 2.

1. Mutation information is provided using different versions of the human reference genome assembly. We recommend using the GRCh38/hg38 assembly, along with the genomic coordinates (i.e., chromosome number: precise position on the chromosome).
2. Alternatively, the mutation information is based on the transcript. This nomenclature includes details about the reference transcript (e.g., NM_007181), the exon where the mutation is located (e.g., exon 23), the coding DNA coordinate (e.g., c.1778), and a description of the nucleotide change (e.g., T>G).
  - Note: A single gene can have multiple transcripts, and therefore different coding DNA coordinates. Always make sure to use the correct transcript ID.
3. Go to Ensembl.org and search for the gene of interest.
4. Copy the assembly ID (written next to the gene name).
  1. Go to SnapGene, select “Import” and “Ensembl sequences” and import gene by pasting the copied ID from Ensembl.
    - Note: Alternative gene visualization tools to SnapGene can be used from this step forward. Please follow instructions of the specific tool.
  2. If the patient-specific sequence is available, align it in SnapGene to verify that the sequence is matching to find the exact position of the mutation:
    - Alignment: Ctrl + F or “Edit”→“Find”→“Find DNA Sequence”.
  3. If patient-specific sequence is not available, add it manually:
    - Go to https://genome.ucsc.edu/and select “Genome Browser”.
    - Select GRCh38/hg38 in human assembly drop-down menu and the genomic position in “Position/Search Term”.
    - The mutated nucleotide is highlighted in yellow. Use zoom out function for better visualization.
    - In the menu on the bottom of the page, enable NCBI RefSeq: pack (or according to the preference) to obtain information on exon/intron, codon, amino acid, and position.
  4. Reduce the length of the target sequence to ± 400 bp from the mutation/target site and create two files for CRISPR reagent design:
    - File that shows the sequence after editing.
    - File that shows the sequence before editing (e.g., WT/MUT sequence).

### 2.2 CRISPR gRNA design and visualization

This step outlines the design of CRISPR/Cas9 gRNAs for *in vitro* validation. We recommend testing two to five gRNAs per target site in primary T cells to identify safe gRNAs with high HDR efficiency (Figure 2).

1. Open SnapGene file containing the patient/wild-type sequence intended for editing.
  - Note: It is important to have the correct sequence for gRNA design as efficient gRNAs for pathogenic variants tend to overlap the mutation site (Mamia et al., 2025).
2. Identify possible PAM sites within the target site by searching for “GG” to highlight available PAM sites (5-NGG-3′) for SpCas9.
  - Note: For homozygous recessive mutations, we recommend limiting the gRNA designs within 10–20 bp from the mutation site to reduce the number gRNAs to validate *in vitro* (Mamia et al., 2025).
  - Note: For heterozygous mutations, we strongly recommend designing gRNAs that overlap the mutation site in the gRNA seed region (i.e., the first 10 nt upstream of the PAM site) to reduce the risk of cutting the healthy allele.
3. gRNA sequence is the 20 nt sequence directly upstream of the PAM site.
  - Note: gRNAs with high likelihood of off-target sites should be eliminated from further testing, using gRNA off-target prediction tools, such as CHOPCHOP (Labun et al., 2021; Labun et al., 2019).
  - Note: Both forward (5’→3′) and reverse (3’→5′) gRNAs can be used (gRNA orientation based on gene orientation), we recommend matching the and single-stranded oligodeoxynucleotide (ssODN) orientation with the gRNA orientation to achieve high on-target editing as a mismatch in these could negatively impact HDR editing (data not shown).
4. Mark Cas9 cut site on the gRNA design (3 bp upstream from the PAM site).
5. Order gRNAs with sgRNA formulation (ready-to-use) or as a crRNA that is annealed 1:1 with a tracrRNA by following the vendor’s instructions.

**FIGURE 2:**
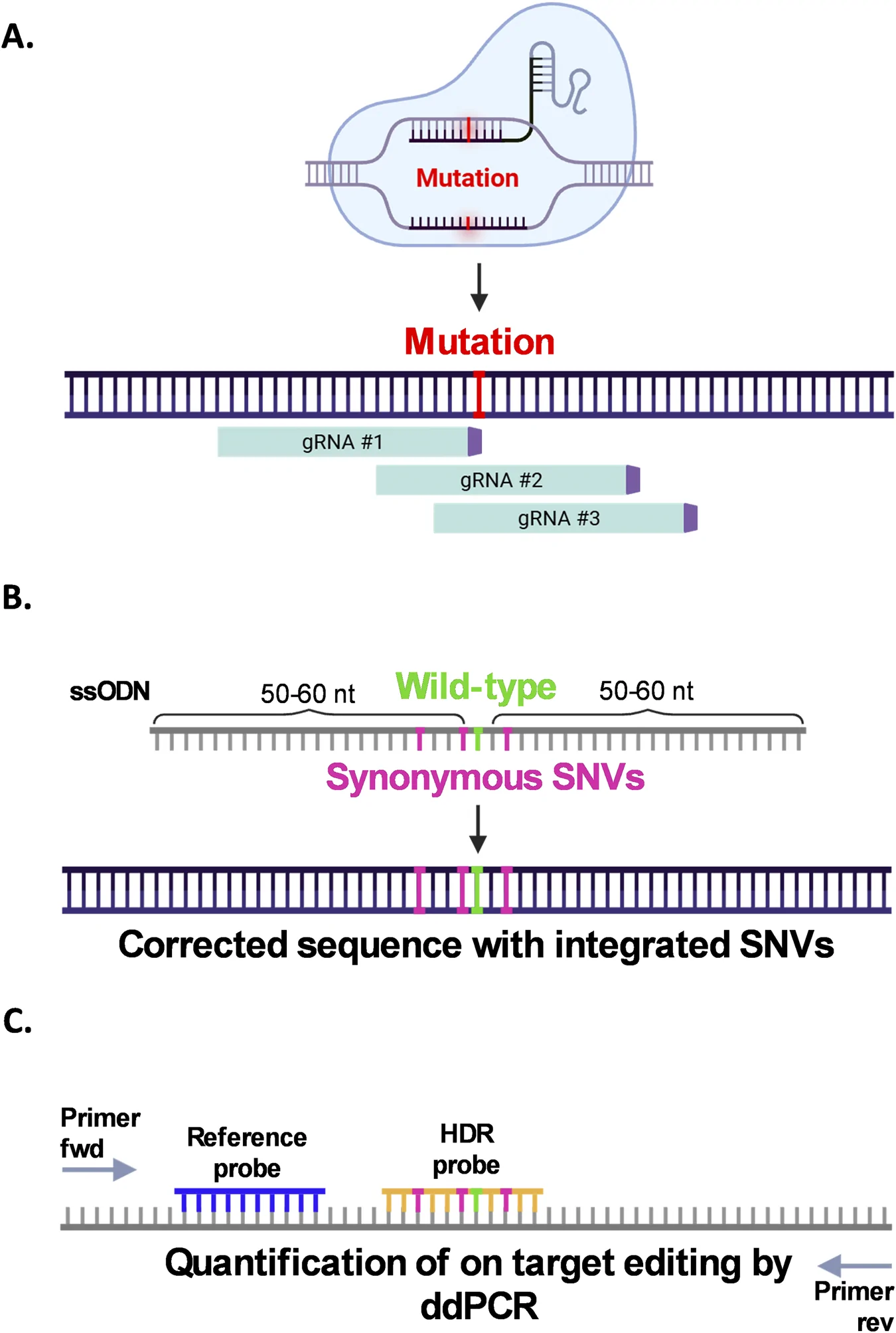
CRISPR/Cas9 reagent design and on-target assessment. **(A)** Schematic representation of the gRNA screening strategy, where multiple gRNAs (green) per target site are designed based on the available PAM sites (purple) within the 100 bp ssODN area. Mutation site is marked with red. **(B)** Schematic representation of the repair strategy, where 100–120 nt ssODNs with ± 60 nt homology arms from the mutation site (red) are used. ssODN design includes correction of the mutation (green) and two to three silent SNVs (pink), enabling identical editing strategy in patients and healthy controls and HDR detection by ddPCR. **(C)** Schematic representation of the ddPCR assay design for HDR detection, where forward and reverse primers amplify a ∼250 bp region surrounding the editing site. Reference probe (blue) binds to unedited sequence outside of the repair template and HDR probe (orange) binds to the edited sequence, which allows for precise quantification of HDR in edited T cells. Abbreviations: guide-RNA (gRNA), protospacer adjacent motif (PAM), single-stranded oligodeoxynucleotide (ssODN), base pair (bp), nucleotide (nt), single nucleotide variant (SNV), droplet digital PCR (ddPCR), homology-directed repair (HDR).

### 2.3 CRISPR ssODN design and visualization

This step outlines the ssODN design steps for HDR-based editing (e.g., mutation correction) at the target site. In order to prevent Cas9 from re-cutting the edited strand (Okamoto et al., 2019; Paquet et al., 2016), we recommend integrating two to three synonymous SNVs into the ssODN design in addition to mutation correction (Figure 2) (Mamia et al., 2025). When adding synonymous SNVs to the repair template design, we recommend placing them at the PAM site and or the gRNA seed region (i.e., the first 10 nt upstream of the PAM site). This strategy also helps to increase accuracy of ddPCR for HDR detection (Mamia et al., 2025) (see Step 8 below).

1. Design 100–120 nt long ssODN for the target site using the following guidelines:
  - The ssODN should center the mutation/target site with 50–60 nt homology arms on both sides surrounding the mutation.
  - The additional two to three SNVs should be synonymous, where the nucleotide change does not change the amino acid sequence.
  - The SNVs should not be located at a highly conserved nucleotide position.
    - Note: Conserved regions can be visualized with UCSC genome browser (https://genome.ucsc.edu/).
  - SNVs should not be in the intron region.
  - SNVs should not affect splicing.
    - Note: Predictions can be performed using SpliceAI (https://spliceailookup.broadinstitute.org/), CADD scoring (https://cadd.gs.washington.edu/score) and Human Splicing Finder Pro (https://genomnis.com/hsf).
  - The SNVs and the mutation/target site should not be more than 10–20 bp apart.
2. Add 2X phoshorothioate (PT) modifications on the 3′ end of the ssODN to improve HDR by preventing the repair template from 3′ endonuclease degradation (Mamia et al., 2025).

### 2.4 ddPCR reagent design to assess on-target HDR editing

This step outlines the design of the reagents for assessing on-target editing by ddPCR (Figure 2). Alternatively, commercially designed kits (e.g., Bio-Rad GED HDR Assay) can be used for assessing on-target editing. We note that ddPCR assay is reliable for HDR detection (Mamia et al., 2025) but does not as accurately capture NHEJ. Alternatively, we recommend assessing on-target editing with NGS-based methods, such as amplicon sequencing, which we have described previously (Mamia et al., 2025; Reint et al., 2021).

1. ddPCR primer design:
  - Use a 300 bp sequence length with ± 150 bp arms from the mutation site for primer design with a chosen software (such as Primerblast, Primer3 etc*.*).
  - Set parameters for primer design: amplicon length at 150–300 bp, primer Tm 54 °C–58 °C.
    - Note: primers should amplify a region long enough to include the repair template entirely as well as the ddPCR probes (reference, HDR and optionally NHEJ).
  - Choose two to three designs and test primers on a 2% agarose gel to confirm primer specificity.
    - Note: To test primers, we recommend validating them using DNA from the target cell type (T cells).
    - Note: We recommend testing different PCR annealing temperatures (e.g., ± 5 °C of the primer Tm) to find optimal PCR conditions (Step 8).
    - Note: consider using a restriction enzyme to reduce unspecific amplification. 2–5 U of restriction enzyme is recommended per sample and is added in the ddPCR mastermix with other reagents (Step 8).
2. General ddPCR probe design guidelines:
  - Primer sequence cannot overlap the probe sequence.
  - Probe Tm should be 3 °C–10 °C higher than primer Tm.
  - GC content should be 30%–80%.
  - Probe length should be < 30 nt, ideally 20 nt. Shorter probes are more efficient at discriminating between single nucleotides.
  - The 5′ end of the probe should not start with a G (this quenches the probe fluorescence)
3. Reference ddPCR probe design:
  - Reference probe should be located outside of the target editing site and ideally outside the repair template.
    - Note: External reference probe within another locus can alternatively be used in the assay. However, we did not find differences in ddPCR readouts when comparing internal and external reference probes (Mamia et al., 2025).
  - Reference probe should be ordered with 5′ HEX and 3′BHQ1 fluorochromes.
    - Note: Other quenching fluorochromes can be used. Follow vendor guidelines.
4. HDR ddPCR probe design:
  - HDR probe should center the mutation/target site and contain the sequence of the edit, not the unedited sequence.
  - HDR probe should be ordered with 5′ FAM and 3′BHQ1 fluorochromes.
5. Optional: NHEJ ddPCR probe design:
  - NHEJ probe should center the Cas9 cut site and contain the sequence of the unedited target.
  - NHEJ probe should be ordered with 5′ FAM and 3′BHQ1 fluorochromes.

## 3 Patient/healthy control PBMC isolation from whole blood using density grade centrifugation

### 3.1 Timing [3 h]

This step describes the process of peripheral blood mononuclear cell (PBMC) isolation from whole blood samples (Figure 3A) collected in sodium heparin tubes. The process results in a clean PBMC population that does not contain plasma, platelets or red blood cells.

1. Pipette 15 mL Lymphoprep per LeucoSep tube and briefly spin down (short spin, 30 s) to move the Lymphoprep below the filter line.
2. Pool 10–20 mL of whole blood from sodium heparin blood collection tubes into a 50 mL Falcon tube.
  - Note: If blood sampling volume is higher, divide blood into multiple 50 mL Falcon tubes.
3. Rinse blood collection tubes with RT PBS and add into the previously prepared 50 mL tube(s).
4. Further fill the 50 mL tube containing the blood up to 40 mL with RT PBS to dilute the blood, mix by pipetting.
5. Pipette diluted blood (40 mL per tube) into the LeucoSep tube by gently dispensing the liquid onto the tube wall.
  - Note: Be careful with pipetting (use low dispensing speed for) to prevent blood from flowing below the filter.
6. Centrifuge LeucoSep tubes for 20 min at 800 g with brakes off (1) and acceleration off (1).
  - Note: The centrifugation takes approximately 30 min to finish.
7. After centrifugation, remove some (∼15–20 mL) of the plasma layer on top with a pipette.
  - Note: This helps to access the PBMC layer better in the next step.
8. Harvest the enriched PBMC cell fraction below the plasma layer by aspirating the cells with a 1,000 μL pipette tip into clean 50 mL Falcon tubes pre-filled with ∼20 mL RT PBS.
  - Note: We recommend that contents of one LeukoSep tube goes into one clean 50 mL tube for optimal washing in the next step.
  - Note: Do not aspirate Lymphoprep below the PBMC layer as it can be toxic for the cells.
9. Fill each tube up to 50 mL with RT PBS and mix gently by pipetting.
10. Centrifuge for 10 min at 100 g with brakes on (9) acceleration on (9).
11. Discard supernatant by aspirating with a pipette.
  - Note: PBMC pellet is large but easily dislodged. Do not discard supernatant by pouring.
12. Resuspend cell pellet gently first in 1,000 μL RT PBS and then fill up to 50 mL. Mix by pipetting.
  - See Troubleshooting Problem one if PBMCs are aggregated and difficult to dislodge by pipetting at this stage.
  - See Troubleshooting Problem two if a high amount of red blood cells are present in the cell pellet at this stage.
13. Centrifuge for 5 min at 300 g with brakes on (9) acceleration on (9).
14. Discard supernatant like previously described and resuspend pellets first in 1,000 μL RT PBS. Pool pellets from tubes together and then fill up to 50 mL PBS.
15. Take an aliquot for counting to record cell count and viability.
  - Note: If PBMC yield is high, cells should be diluted in PBS before counting to ensure accurate measurement.
16. Centrifuge cells for 5 min at 300 g with brakes on (9) acceleration on (9).
17. Discard supernatant and proceed to cryopreservation (Step 3) or cell culture (Step 4).

**FIGURE 3:**
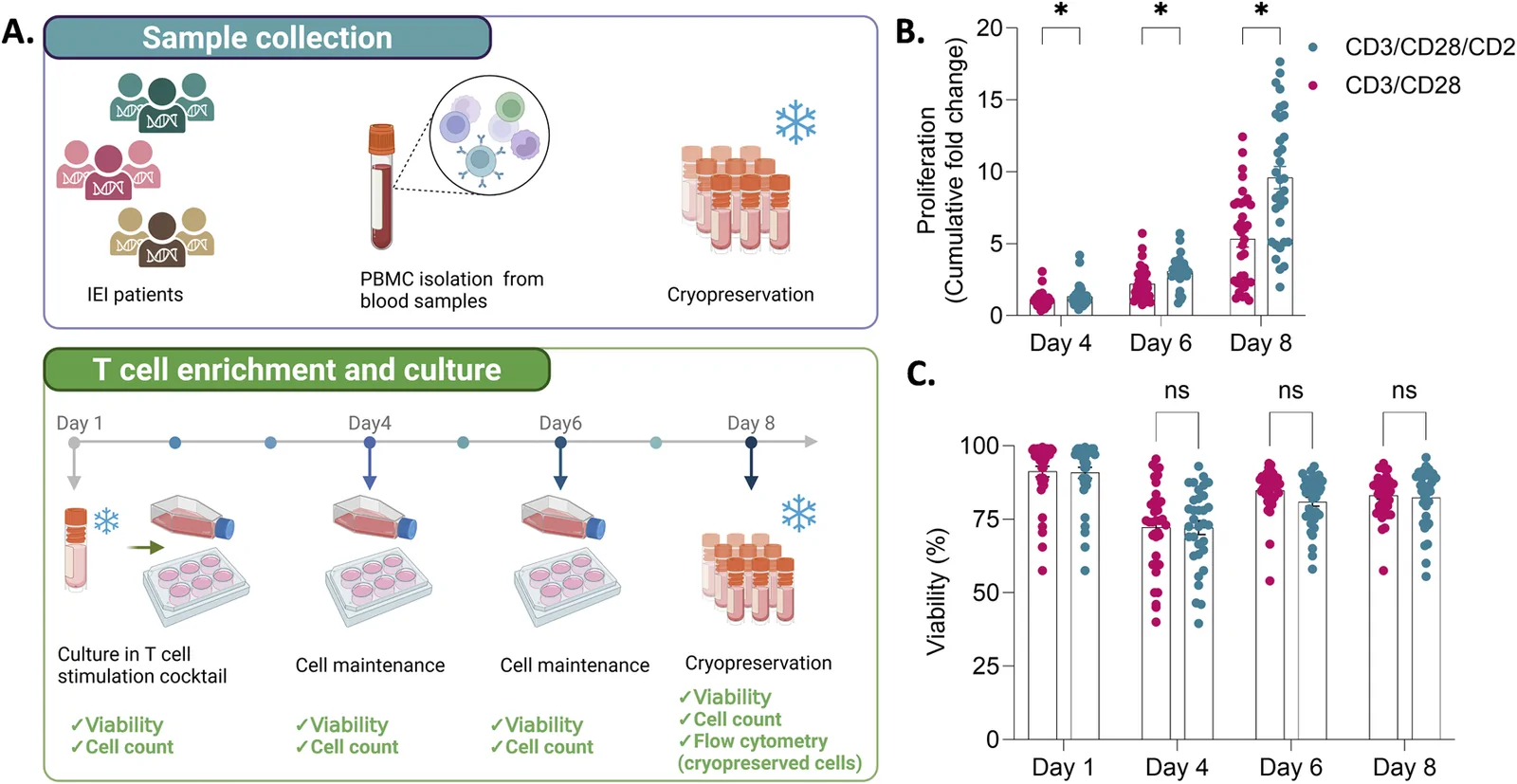
Overview of the T cell culture platform. **(A)** Schematic of the eight-day T cell enrichment and culture workflow. In brief, PBMCs isolated from blood are cryopreserved, then thawed and cultured with IL-2, IL-7, IL-15, and CD3/CD28 or CD3/CD28/CD2 stimulation. After 3 days, medium is refreshed with interleukins on days 4 and 6. Cells are cryopreserved on day 8 for flow cytometry. Viability and cell counts are recorded on days 1, 4, 6, and 8. **(B)** Viability and **(C)** cumulative fold expansion relative to day 1 of the cells derived from 33 IEI patients. Data are presented as mean ± SEM. Statistical analyses were performed in GraphPad Prism using the Wilcoxon matched-pairs signed rank test (nonparametric, paired design). P values were adjusted for multiple comparisons using the Holm–Šidák method, with a significance threshold of α = 0.05. Abbreviations: peripheral blood mononuclear cell (PBMC), Interleukin (IL).

## 4 PBMC cryopreservation

### 4.1 Timing [30 min]

This step describes PBMC cryopreservation for long-term storage at −150 °C prior to thawing the cells for editing experiments. We recommend cryopreserving a minimum of five million cells/vial (patients/small sampling volumes) and a maximum of 30 million cells/vial (healthy controls/large sampling volumes) for optimal cell recovery.

1. Based on the cell count, prepare to freeze 5–30 million cells per cryovial. Freezing volume per vial is 1 mL unless cell number is < 5 million, which is when reduced freezing volumes can be used (e.g., 0.5 mL).
2. Label cryovials with appropriate information, including donor/patient number, date and number of cells per vial.
3. Resuspend cell pellet from Step 2.17 first in 1 mL of 4 °C CryoStor freezing medium using a 1,000 μL pipette, then fill up to a desired volume.
  - Note: DMSO in freezing medium is toxic for the cells. When freezing cells, perform resuspension quickly while remaining gentle with pipetting.
  - Note: Resuspending cell pellet with a 1,000 μL tip first is recommended to ensure cells are in single-cell suspension in the freezing medium for optimal recovery.
4. Add 1 mL cell suspension per cryovial, close caps tightly and place vials into CoolCell container.
5. Move container immediately to −80 °C for o/n storage.
  - Note: Do not store cells at −80 °C for more than 1–4 days to ensure optimal recovery.
  - Note: Do not stack CoolCell containers on top of each other at −80 °C to ensure equal freezing between the containers.
6. Transfer cryovials to −150 °C for permanent storage.

## 5 PBMC thawing and cytokine stimulation (day 1)

### 5.1 Timing [1 h]

This step describes the thawing and stimulation of cryopreserved PBMCs. This step is not necessary if PBMCs are cultured directly after isolation as described in Step 2. PBMCs are stimulated with a cytokine cocktail, which we have shown previously to promote CD4^+^ and CD8^+^ T cell activation and proliferation (Figure 3A) (Mamia et al., 2025).

1. Transfer cells from −150 °C onto dry ice.
2. Pipette 10 mL of RT ImmunoCult T cell expansion medium into a 15 mL Falcon tube per each cryovial to be thawed.
3. Thaw cells in a 37 °C water bath for approximately 2 min until a pea-sized frozen part is left.
  - Note: do not immerse cryovials fully in water bath to prevent water from getting into the cap as this could result in contamination.
4. Spray cryovial with 70% ethanol, wipe, and transfer into sterile laminar hood.
5. Gently pipette 1,000 μL RT ImmunoCult T cell expansion medium into the vial and pour vial contents into the 15 ml Falcon pre-filled with 10 mL RT ImmunoCult T cell expansion medium.
  - Note: Do not pipette cells during this step. When pouring cells, make sure vial contents flows directly into the thawing medium and not the wall of the tube.
6. Add 1,000 μL RT ImmunoCult T cell expansion medium into the cryovial to wash out remaining cells and pool with the previous.
7. Centrifuge cells at 300 g for 5 min.
8. Discard supernatant, resuspend pellet first in 1 mL with a 1,000 μL tip with pre-warmed Immunocult T cell expansion medium supplemented with anti 15 μL/mL CD3/CD28 or 15 μL/mL CD3/CD28/CD2 and IL-2 (120 U/mL), IL-17 (3 ng/μL) and IL-15 (3 ng/μL). From here onwards, this medium is referred to as ‘T cell stimulation medium’. Then add up to 1–10 mL depending on the size of the pellet.
  - Note: cells should be at a minimum of one million cells/mL at the time of counting to avoid unnecessary centrifugation step.
9. Mix gently by pipetting. Take an aliquot for counting.
10. Record cell viability and amount of live cells/mL.
11. Dilute cells into one million live cells/mL with pre-warmed T cell stimulation medium.
  - Note: We have compared 2 T cell stimulation cocktails (CD3/CD28 or CD3/CD28/CD2 in healthy controls and 33 IEI patients (Figure 2).
12. Pipette cell suspension into an appropriate cell culture plate/flask and incubate cells at 37 °C/5% CO_2_ for 3 days without disturbing them.
  - Note: We advise using tissue-culture treated cell culture plates/flasks to promote beneficial cell-to-cell communication between T cells and macrophages during early stages of PBMC stimulation.
  - Note: The choice of cell an appropriate cell culture flask depends on the cell count. We recommend following the guidelines below prevent drying of cells while ensuring proper gas exchange in the flask:
    - < 1 million: One well in 12-well plate
    - < 2 million: One well in 6-well plate
    - two to four million: T-25 flask standing upright
    - 4–6.5 million: T-25 flask lying down
    - 6.5–10 million: T-75 flask standing upright
    - 10–15 million: T-75 flask lying down
    - 15–30 million: T-175 flask standing upright
    - >40 million: T-175 flask lying down

## 6 PBMC dilution (day 4)

### 6.1 Timing [15 min]

This step describes dilution of the previously stimulated PBMCs. If there are enough cells on day 4, this step is optional, and one can proceed directly to nucleofection for gene editing. However, when performing large experiments or working with little starting material, we recommend performing this step prior to nucleofection to expand cells further.

1. Dilute cells at 0.25–0.5 million cells/mL or 1:1 in volume on day 4 by adding pre-warmed T cell stimulation medium (only IL-2, IL-17 and IL-15) without CD3/CD28 or CD3/CD28/CD2.
  - Note: Keep cells in the same flask at this step as they are partially adhered at this stage and expand more efficiently if the cell culture vessel is not changed.
  - Note: Cell counts (especially for patients) at this stage are not reliable due to the partially adhered cells.
  - Note: Cell dilution factor depends on the patient/donor and should be assessed case-by-case. We note that patient cells often expand more slowly compared to healthy controls, where healthy controls can be diluted to 0.25 million cells/mL without issues.
  - See Troubleshooting Problem three if cell viability is low on day 4–8 of the protocol.
2. Incubate cells at 37 °C/5% CO_2_ for 2 days and repeat dilution on day 6 and 8 if necessary.
  - Note: It is ideal to nucleofect cells between day 4–8 of the protocol to achieve high HDR editing. After day 8, HDR levels decrease rapidly, as we have previously demonstrated (Mamia et al., 2025).
  - See Troubleshooting Problem four if there are not enough cells for nucleofection on day 4 of the protocol.

## 7 T cell nucleofection (day 4–8)

### 7.1 Timing [1–3 h depending on the number of samples]

This step describes the nucleofection of previously stimulated PBMCs. We recommend performing the nucleofection between day 4–8 of the PBMC stimulation protocol to ensure high HDR levels in the samples (Mamia et al., 2025). Edited cells can be collected 4 days after nucleofection or expanded further.

1. Pre-program the Lonza 4D electroporation unit for 16-well strip or 96-well plate depending on the size of the experimental setup.
2. Prepare cell culture plates by adding Immunocult T cell expansion medium supplemented with IL-2 (250 U/mL) into the wells. From here onwards, this medium is referred to as ‘T cell recovery medium’. Incubate at 37 °C/5% CO_2_ while proceeding with the protocol.
  - Note: Cell culture plates and working volumes depend on the number of cells per nucleofected sample. As low as 0.1 million cells per sample can be nucleofected (Mamia et al., 2025) but due to the fragility of patient cells, we recommend opting for 0.five to one million cells per nucleofected sample.
    - 0.1–0.25 million cells: 96-well plate with a final volume of 200 uL
    - 0.5–0.7 million cells: 48-well plate with a final volume of 400 uL
    - 0.eight to one million cells: 24-well plate with a final volume of 500 uL
3. Prepare CRISPR RNPs for nucleofection:
  - Mix 100 pmol sgRNA or 1:1 annealed crRNA-tracrRNA and 61 pmol Cas9 nuclease per reaction by pipetting or vortexing gently.
    - Note: If multiple different experimental conditions are tested, we recommend preparing the individual RNP reactions in a 96-well plate and sealing the plate with foil.
    - Note: RNPs can be made for several replicas in one reaction (i.e., in 1.5 mL tubes) if experimental design allows.
  - Briefly spin the plate/tube down and incubate at 37 °C for 15 min.
  - Transfer reaction on ice until 100 pmol repair template/reaction is added.
    - Note: repair template should be added when cells are ready for nucleofection to prevent RNP degradation.
4. Prepare cells for nucleofection:
  - Harvest cells from cell culture flasks and transfer into a 50 mL Falcon tube
    - Note: Additional PBS washing in the flask can be performed to harvest as many cells as possible.
  - Centrifuge cells at 300 g for 5 min.
  - Discard supernatant by pipetting and resuspend cell pellet in 10–50 mL PBS depending on the number of cells.
  - Mix and take an aliquot for counting.
  - Count the volume of cell suspension needed to obtain the desired number of cells for nucleofection (0.one to one million cells per nucleofected sample).
  - Transfer desired volume into a new 15 or 50 mL tube and centrifuge at 300 g for 5 min.
    - Note: We recommend adding the repair template to the RNP mix at this stage.
  - Discard supernatant by pipetting and resuspend cell pellet in P3 nucleofection buffer with 20 μL buffer per nucleofected sample.
    - Note: Depending the nucleofection buffer (we recommend Lonza Primary cell P3 nucleofection buffer for primary T cells), it is advisable to work fast as some buffers can be toxic for primary cells.
    - Note: It is important that cells are in single cell suspension in the nucleofection buffer to ensure successful nucleofection and editing levels.
5. Mix cells with RNPs:
  - Add 20 μL of cell suspension per sample into the previously prepared RNPs.
    - Note: If multiple different experimental conditions are tested in the experiment, we recommend adding the cells into the RNPs on a 96-well plate.
    - Note: Several replicas can be made in one reaction (1.5 mL tubes) if the experimental design allows.
  - Mix gently but thoroughly to obtain a homogenous single-cell suspension.
6. Perform nucleofection:
  - Using a multichannel pipette (RNPs in a 96-well plate) or an electronic pipette (RNPs in 1.5 mL tubes), transfer 20–25 μL cell suspension per electroporation cuvette well.
    - Note: Cell volume can be adjusted according to the volume of RNPs (RNP volume is usually 3–5 μL per sample depending on the reagent concentrations) but should not exceed 30 μL.
    - Note: Pipette carefully when transferring cell-RNP suspension into the nucleofection wells as bubbles may disturb the procedure and affect cell viability and editing outcomes.
  - Nucleofect selected wells using pulse code ‘EO-115’ and solution ‘Primary Cell P3’.
    - Note: Different pulse codes and solutions may be used for different experiments. This setup works for previously stimulated healthy donor and patient T cells.
  - Immediately after nucleofection, pipette 85 μL pre-warmed T cell recovery medium into each well.
    - Note: Do not pipette cells up and down as they are fragile at this stage.
  - Incubate cells at 37 °C/5% CO_2_ for 15 min.
7. Transfer nucleofected cells into cell culture plates:
  - After incubation, add 50 μL of T cell recovery medium into each well.
  - Transfer cells into previously prepared cell culture plates with a multichannel pipette.
  - Gently move the plates in a ‘criss-cross’ movement to distribute cells evenly without pipetting.
  - Incubate cells at 37°C/5% CO_2_ for 24 h.

## 8 T cell maintenance after nucleofection (between day 5–12)

### 8.1 Timing [30 min]

This step describes the maintenance protocol for nucleofected T cells. We recommend performing the first dilution/splitting step 24 h after nucleofection and the second step 72 h after nucleofection. We recommend collecting cells 4 days after nucleofection (day 8 of the platform) as at this point HDR editing levels have stabilized (Mamia et al., 2025) unless the edited cells have a growth advantage/disadvantage, which requires a timepoint experiment to determine the optimal time for sample collection.

1. 24 h after nucleofection, dilute/split cells by adding pre-warmed T cell recovery medium onto the cells.
  - Note: Dilution/splitting ratio depends on the donor and the well size. Healthy controls can typically be split at 1:2 to 1:3 at this stage as the cells tend to grow fast. Depending on the patient, cells can be diluted (i.e., add medium) or split at 1:1 to 1:2.
  - Note: When splitting cells, make sure to dislodge T cell colonies into single-cell suspension by pipetting gently. When working with very fragile patient cells where only dilution is performed, keep cells at colonies at this stage to avoid shearing stress on the cells.
  - Note: Optimal cell density after dilution/splitting at this stage is 0.four to one million cells/mL depending on the donor. Cells density before the next splitting/dilution (72 h after nucleofection) should not exceed 2–2.5 million cells/mL to ensure high cell viability.
  - Note: Keep in mind the recommended working volumes in a given well and transfer cells into a larger plate when needed:
    - 96-well plate: 100-200 μL
    - 48-well plate: 200-400 μL
    - 24-well plate: 500-1000 μL
    - 12-well plate: 1000-2000 μL
2. 72 h after nucleofection, repeat cell dilution/splitting while maintaining appropriate T cell density.
  - Note: At this stage, T cell colonies are typically large and dislodging them by gentle pipetting is recommended.
3. 96 h after nucleofection, harvest cells for downstream analyses or cryopreservation.
  - Note: Cells can be further expanded by continuing to culture them in T cell recovery medium, which is added every second day.
  - Note: If cells are expanded, we recommend to re-stimulate them on day 10–12 of the platform with full T cell stimulation medium (CD3/CD28 or CD3/CD28/CD2 and IL-2, IL-17 and IL-15). T cells are then seeded at one million cells/mL as described in Step 4. After re-stimulation, cells should be diluted with T cell stimulation medium without CD3/CD28 or CD3/CD28/CD2 every 2–3 days at 0.25–0.4 million cells/mL depending on the donor/patient.

## 9 On-target editing assessment by ddPCR (day 8 or as preferred)

### 9.1 Timing [8 h]

This step describes the process of sample preparation for ddPCR to detect on-target editing in the samples. We note that the assay is more reliable when in addition to mutation correction, two to three additional synonymous SNVs have been integrated into the repair template design (see Step 1.3). This ensures that the HDR probe can discriminate between an unedited and edited sequence, which can be more challenging with only a single nucleotide correction. Alternatively, commercially designed kits (e.g., Bio-Rad GED HDR Assay) can be used, which have been optimized for single nucleotide correction detection.

1. Extract gDNA from cells as according to kit instructions.
  - Note: we recommend 0.five to one million cells/sample to achieve high gDNA yield.
  - Note: gDNA samples can be stored at −20 °C indefinitely until running the ddPCR assay.
2. Measure gDNA concentration using Qubit or other sensitive dsDNA concentration measurement kit.
3. Normalize gDNA concentration across samples to 8 ng/μL with nuclease-free ddH_2_O.
  - Note: If DNA stock concentration is < 8 ng/uL, it is possible to use lower dilutions for the assay at as low as 1 ng/uL as long as all samples have been normalized to the same concentration.
4. Pipette 8 μL of DNA dilution per well on a 96-well semi-skirted ddPCR plate.
  - Note: ddPCR DNA dilutions can be stored at −20°C until running the assay.
5. Thaw ddPCR primers, FAM and HEX probes and ddPCR supermix (see design of custom ddPCR reagents in Step 1.4) in room temperature.
  - Note: ddPCR probes are light-sensitive and have a longer shelf-life when they are protected from light.
6. Prepare ddPCR master mix for HDR/NHEJ assay in a DNAse/RNAse free 1.5 mL tube:
  - Calculate the volume of reagents needed as according to Table 1.
  - Add nuclease-free ddH_2_O, ddPCR primers, reference probe, HDR/NHEJ probe and mix by vortexing.
  - Vortex ddPCR supermix vigorously to solubilize any precipitated crystals.
  - Add ddPCR supermix to the reagents and mix by vortexing.
7. Add 12 μL ddPCR mastermix per sample into 96-well semi-skirted ddPCR plate with the diluted gDNA.
  - Note: If there are empty wells in the column, fill them up with 20 μL of water.
8. Seal the plate, mix by vortexing. Briefly spin down.
9. Generate droplets using Automated Droplet Generator as according to manufacturer’s instructions.
10. Place pierceable foil on droplets and perform heat sealing.
11. Run PCR according to Table 2.
  - Note: the program takes about 3.5 h to finish, finished plate can be stored at 4°C o/n prior to reading the plate.
12. Read droplets in Droplet Reader.
13. Analyze data according to experimental design using QX Manager.
  - See Troubleshooting Problem five if HDR editing is low in the samples (0%–10% HDR).
  - See Troubleshooting Problem six if NHEJ editing is low in knockout experiments (i.e., when repair template is not used).

**TABLE 1:** ddPCR PCR reagents.

Reagent	Stock concentration	Volume per reaction (µl)
ddPCR supermix (no dUTP)	N/A	10
Primer fwd	100 μM	0.18
Primer rev	100 μM	0.18
ddPCR References probe	100 μM	0.1
ddPCR HDR/NHEJ probe	100 μM	0.1
milliQ H_2_O	N/A	1.44
DNA	8 ng/μL	8

**TABLE 2:** ddPCR cycling conditions.

Step	Temperature	Time	Cycles
Initial denaturation	95 °C	10 min	1
Denaturation	94 °C	30 s	42X
Annealing	56 °C	3 min	
Final extension	98 °C	10 min	1
Hold	4 °C	Indefinitely	1

## 10 Flow cytometry profiling of cells from the platform (day 8 or as preferred)

### 10.1 Timing [3 h]

This step describes the process of sample preparation and staining for flow cytometry to study and characterize T cell populations present in the edited cells at the end of the protocol.

1. Centrifuge cells at 400 g for 5 min at 4 °C.
  - Note: we recommend 0.five to one million cells/sample for flow staining.
2. Discard supernatant and resuspend cells in 200 µl cold PBS.
3. Transfer cell suspension into a 96-well V bottom plate.
4. Centrifuge cells at 400 g for 5 min at °C.
5. While centrifuging, prepare 50 µl of 1/500 dilution of Live/Dead Violet dye in PBS with 1/20 dilution of human Fc block for each sample (e.g., 2 µl Live/Dead +50 µl Fc block per 1,000 µl PBS for 20 samples).
  - Note: Do not re-freeze the thawed stock dye solution, discard any leftover.
6. Discard the supernatant.
7. Add 50 µl of the Live/Dead Dye + Fc block mix to each sample. Mix well. Stain for 30 min 4 ° C on ice and dark.
8. After the 30 min of live/dead staining + Fc block, add 100 µL of PBS to each sample.
9. Centrifuge at 400 g for 5 min at 4 °C.
10. While centrifuging, prepare the antibody mix (Table 3). Add 41.75 µL of the flow staining buffer to the antibody mix to achieve staining volume of 50 µL.
11. Discard the supernatant and add 50 µL of antibodies mix to each sample. Mix well.
12. Stain for 30 min 4 °C on ice and dark.
13. While staining cells, prepare compensation controls:
14. Prepare compensation controls:
  - Completely resuspend the AbC™ Total Compensation capture beads (Component A) and negative beads (Component B) by gently vortexing for 10 s before use.
  - Label flow tubes for each fluorochrome and additional tube for unstained control.
  - Add one drop of AbC™ Total Compensation capture beads (Component A) to each tube.
  - Add 1 µL of each antibody conjugate to the AbC™ Total Compensation capture bead suspension in the designated tube and mix well. Make sure to deposit the antibody directly to the bead suspension. Incubate for 15 min at RT, protected from light.
  - Add 3 mL of PBS to sample tubes. Centrifuge for 5 min at 250 g.
  - Carefully remove the supernatant from tubes and resuspend the bead pellet by adding 0.5 mL of PBS to sample tubes.
  - Add one drop of negative beads (Component B) to each of the tubes and mix well.
  - Vortex tubes before analysing using flow cytometry to increase percentage of singlet beads.
15. After the antibody staining, add 150 µL of cold flow staining buffer to each sample.
16. Centrifuge at 400 g for 5 min 4 °C.
17. Discard supernatant.
18. Resuspend cells in 200 µL of cold flow staining buffer.
19. Transfer samples into flow tubes.
20. Analyze the samples in LSRII (BD Bioscience) or other flow cytometry instrument.

**TABLE 3:** Antibodies and their dilutions used for staining and flow cytometry.

Specificity	Clone	Fluorochrome	Dilution	Volume per sample (µL)
CD3	UCHT1	Alexa fluor 488	1:200	0.25
CD62 L	DREG-56	PerCP-Cy5.5	1:50	1
CD19	HIB19	Pacific blue	1:200	0.25
CD14	M5E2	Pacific blue	1:200	0.25
CD56	MEM-188	Pacific blue	1:200	0.25
CD11c	B-ly6	V450	1:200	0.25
CD8a	RPA-T8	Brilliant violet 570	1:100	0.5
PD-1	EH12.1	PE-CF594	1:25	2
CD45RA	HI100	PE-Cy7	1:50	1
TIM-3	7D3	Alexa fluor 647	1:25	2
CD4	SK3	APC-H7	1:100	0.5

## 11 Cryopreservation of corrected cells

### 11.1 Timing [30 min]

This is the final step of the protocol, where edited cells from the platform are cryopreserved for long-term storage. We recommend cryopreserving a minimum of one million cells/vial (e.g., for later flow cytometry experiments) and a maximum of 30 million cells/vial (e.g., for later experiments requiring cell culture) for optimal cell recovery.

1. Follow cryopreservation steps as described previously for PBMC cryopreservation in Step 3.
  - Note: We recommend following the guidelines below when thawing the cells for further assessments:
    - Flow cytometry: Thaw cells as described in Step 4 at one million cells/mL in ImmunoCult™-XF T Cell Expansion Medium supplemented with 250 U/mL IL-2 and allow to rest o/n at 37°C/5% CO_2_ before proceeding with staining and flow cytometry.
    - Re-stimulation for further experimentation: Thaw cells as described in Step 4 at one million cells/mL in full T cell stimulation medium and allow to rest for 2–3 days before further expansion or intended experiment.

## 12 Results

### 12.1 Expected outcomes

Following the instructions of this protocol, we expect that by the end of the platform (day 8 and beyond) CD3^+^ T cells account for ≥80% of all cells, where frequencies of CD4^+^ and CD8+ T cells depend on the individual (Mamia et al., 2025). Under the PBMC culturing conditions, we expect cells to proliferate, where starting with very low PBMC quantity (one to five million) will result in a robust proliferation and optimal viability (Figures 3B,C). Furthermore, under these culture conditions, we also expect to observe T cells with low exhaustion levels and that central and effector memory T cells persist in the culture (Figures 4, 5). Finally, we expect to see up until 50% HDR in the edited T cells with optimized CRISPR/Cas9 reagent design, and up to 80% when NHEJ inhibitors are used directly after nucleofection (Mamia et al., 2025). However, we note that when working with patient cells with diverse pathogenic mutations, individual variation in T cell proliferation, viability, exhaustion/phenotype and editing should be expected.

**FIGURE 4:**
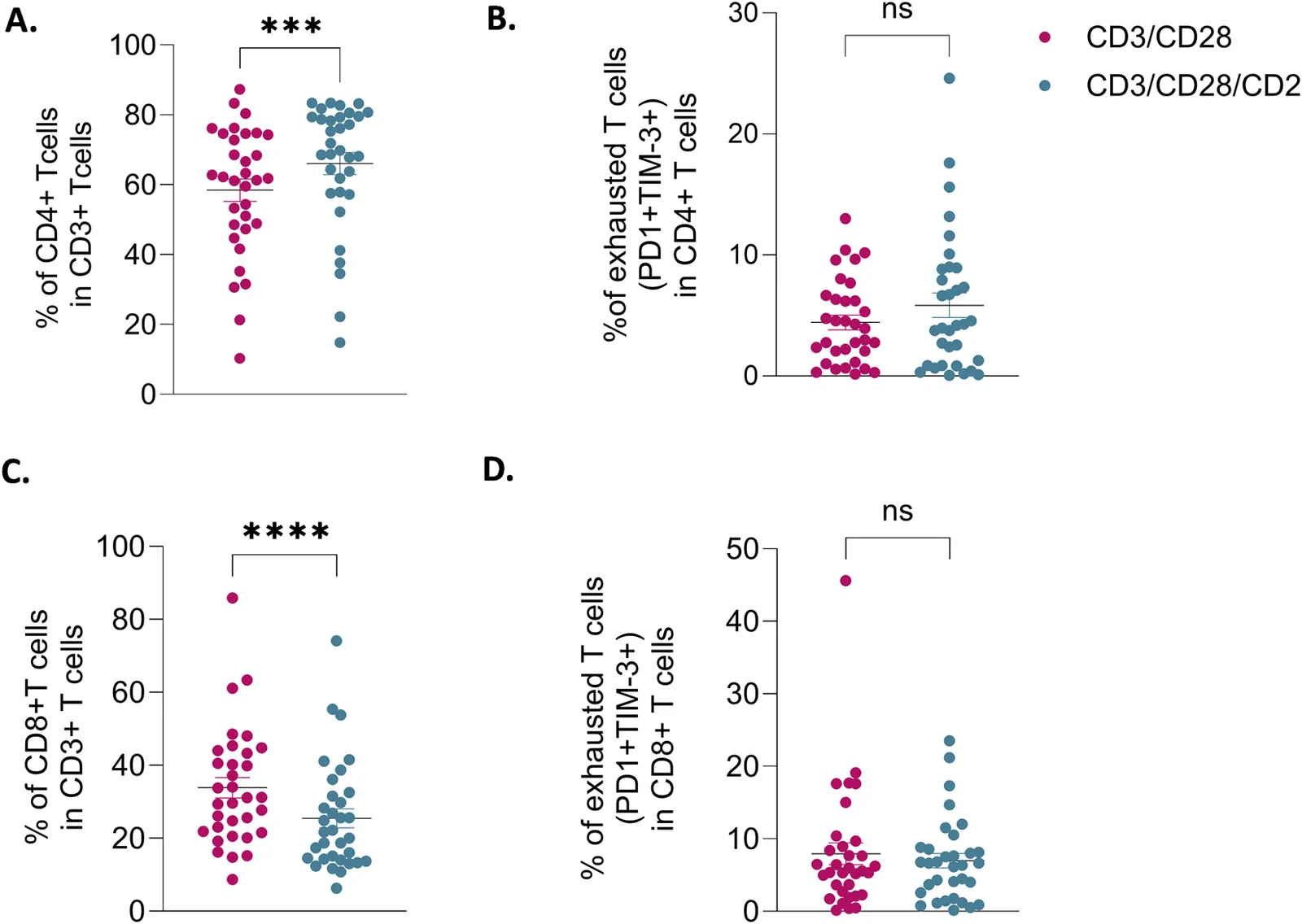
Assessment of T cell exhaustion. Cells generated from cells from 33 IEI patients as described in Figure 2A were cryopreserved on day 8 of the PBMC stimulation and cell culture. The cells were thawed and analyzed by flow cytometry **(A)** CD4^+^ cells among CD3^+^ T cells. **(B)** Exhausted (PD-1^+^TIM-3^+^) cells among CD4^+^ T cells. **(C)** CD8^+^ cells among CD3^+^ T cells. **(D)** Exhausted (PD-1^+^TIM-3^+^) cells among CD8^+^ T cells. Data are presented as mean ± SEM. Statistical analyses were performed in GraphPad Prism using the Wilcoxon matched-pairs signed rank test (nonparametric, paired design). P values were adjusted for multiple comparisons using the Holm–Šidák method, with a significance threshold of α = 0.05. Abbreviations: inborn error of immunity (IEI), peripheral blood mononuclear cell (PBMC).

**FIGURE 5:**
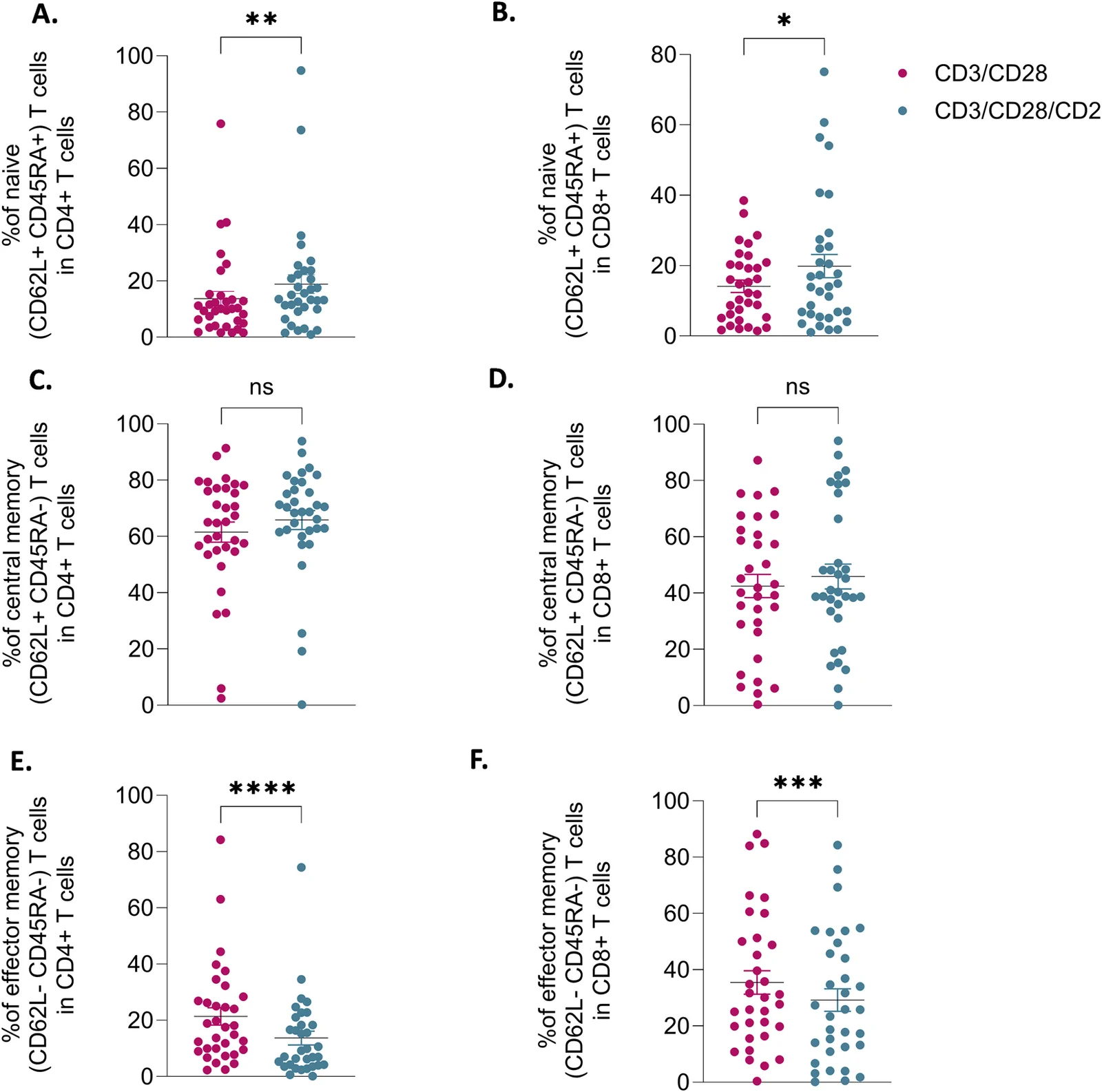
Assessment of phenotype of T cells. Cells generated from cells from 33 IEI patients as described in Figure 2A were cryopreserved on day 8 of the PBMC stimulation and cell culture. The cells were thawed and analyzed by flow cytometry. **(A)** Naïve (CD62L^+^CD45RA^+^) cells among CD4^+^ T cells **(B)** Naïve (CD62L^+^CD45RA^+^) cells among CD8^+^ T cells, **(C)** Central memory (CD62 L^+^CD45RA^-^) cells among CD4^+^ T cells, **(D)** Central memory (CD62L^+^CD45RA^-^) cells among CD8^+^ T cells, **(E)** Effector memory (CD62L^−^CD45RA^-^) cells among CD4^+^ T cells and **(F)** Effector memory (CD62L^−^CD45RA^-^) cells among CD8^+^ T cells. Data are presented as mean ± SEM. Statistical analyses were performed in GraphPad Prism using the Wilcoxon matched-pairs signed rank test (nonparametric, paired design). P values were adjusted for multiple comparisons using the Holm–Šidák method, with a significance threshold of α = 0.05. Abbreviations: inborn error of immunity (IEI), peripheral blood mononuclear cell (PBMC).

Limitations: There are certain limitations to the study, which are discussed here. First, CRISPR/Cas9 gRNA design using SpCas9 is limited by the PAM (5-NGG-3′) availability at the target sequence. If SpCas9 PAM sites are not available, consider using another source of Cas9 (e.g., SaCas9), engineered Cas9 with wider PAM availability (e.g., SpRY-Cas9) or another Cas variant (e.g., Cas12a). Second, it is not possible to integrate synonymous SNVs in the repair template for noncoding mutations as they would change the sequence of the transcribed RNA and likely affect its functions in the cell. However, not having synonymous SNVs in the repair template design can result in reduced HDR editing and issues in accurate HDR detection by ddPCR. To mitigate this, we recommend considering alternative HDR-based assays (e.g., amplicon sequencing) and using other HDR enhancing strategies, such as NHEJ inhibition (Mamia et al., 2025). However, we advise assessing potential safety issues (e.g., cell viability, chromosome arm loss, etc*.*) on a case-by-case basis when using NHEJ inhibitors to boost HDR. Third, there may be individual variation in T cell response to cytokine stimulation presented in the protocol. We anticipate that mutations that affect cytokine signalling (IL-2, IL-7, IL-15) and co-stimulation (CD3/CD28 or CD3/CD28/CD2) require an alternative and user-defined stimulation cocktail to promote cell proliferation.

### 12.2 Troubleshooting

Problem 1: PBMCs are aggregated and difficult to pipette into single-cell suspension for counting and cryopreservation.

Solution: Strain cells through a 40 μM cell strainer to ensure cells are in single cell suspension before counting and cryopreservation to remove the aggregates.

Problem 2: A high amount of red blood cells are present in the PBMCs after density grade centrifugation.

Solution: A red blood cell lysis buffer can be used to eliminate contaminating red blood cells at this stage. Use the reagent of choice and follow the vendor’s guidelines. If the red blood cells are present but they do not significantly affect the PBMC pellet size, the lysis step is not necessary.

Problem 3: PBMC viability is poor (<70%) on Day 4–8 after thawing despite high cell viability upon thawing (>90%).

Solution: If cell viability is drastically decreased upon cell culture and persist by day 8 of the platform, check if the mutation alters cytokine signalling and the subsequent responses to cytokines used in this protocol, as this could result in reduced cell viability and proliferation. When necessary, find alternative T cell stimulation cocktail to promote T cell proliferation from PBMCs. If the mutation does not point to abnormal responses to cytokines, cytokines used in this protocol can be titrated to preserve cell viability. However, we note that this can result in reduced cell proliferation.

Problem 4: There are too few cells to nucleofect on day 4 of the T cell platform.

Solution: If there are not enough cells for nucleofection on day 4 of the cytokine stimulation, dilute cells on day 4 as instructed in Step 5 and repeat cell dilution again on day 6 if necessary. Alternatively, as low as 0.1 Million cells/nucleofected sample can be used, as described in Step 6 and our previous work (Mamia et al., 2025).

Problem 5: HDR editing is low.

Solution: Low HDR editing (0%–10%) can result from several factors, including low cell viability prior to and after nucleofection and competition between HDR and NHEJ as DNA repair mechanisms after Cas9-mediated double-strand break. If cell viability is low (<70%) during nucleofection, cells will typically not survive the nucleofection. Consider trying a different nucleofection code for T cells (consult nucleofector vendor), a different nucleofection instrument (e.g., MaxCyte) or an alternative nucleofection buffer. If cell viability is high (>80%) after nucleofection, it is possible that NHEJ editing is the dominant DNA repair pathway, resulting in low HDR editing. To mitigate this, NHEJ inhibitors, can be used to mitigate this issue. NHEJ inhibitors that have been demonstrated to boost HDR in primary T cells include KU0060648 used at 0.5 μM final concentration for the first 24 h after nucleofection (Mamia et al., 2025). As NHEJ inhibitors also inhibit cell proliferation, reduce the concentration of KU0060648 24 h after nucleofection by pipetting half of the medium out and replacing it by fresh T cell recovery medium. T cells do not need to be centrifuged during the compound dilution step as they typically sediment in large colonies at the bottom of the cell culture wells during this stage.

Problem 6: NHEJ editing is low when the desired editing outcome is a knockout.

Solution: If the gRNA for the experiment has been tested to result in NHEJ, it is possible that the edited cells have a selective disadvantage in cell culture. This is especially likely if the target gene is important for cell survival and proliferation. In that case, it is possible that cells with a successful knockout disappear in culture over time and disappear below the detection line. To mitigate this, we advise performing a time course experiment for all new loci to assess an optimal timepoint for collecting the edited cells.

## 13 Discussion

We describe a non-viral, HDR-based CRISPR/Cas9 platform for precise correction of SNVs in primary human T cells, designed with an emphasis on standardization and modular workflow architecture. By integrating locus-specific components (gRNA, ssODNs, and ddPCR-based on-target editing detection reagents) into a standardized T cell editing pipeline, the system enables reproducible genome modification across multiple donors and genomic loci.

A defining feature of this platform is the separation of locus-specific reagent optimization from a fixed cellular process. Once the custom editing reagents are validated, the downstream T cell culture and editing workflow remains unchanged. This modular structure facilitates the implementation of new genomic targets without the need to redesign the cellular platform, thereby increasing the speed of research and enhancing the scalability and translational applications of this technology.

Importantly, the optimized culture conditions incorporated within the editing platform consistently maintain low levels of T cell exhaustion while supporting the persistence of memory T cell populations across diverse patient samples. This characteristic further underscores the broad applicability of the protocol. In addition, the platform achieves high cell viability and robust editing efficiencies of up to 80% across loci and donors by the end of the workflow.

At the same time, biological parameters intrinsic to primary T cells influence editing outcomes. HDR efficiency is shaped by cell cycle phase, DNA repair pathway activity, and inter-donor variability, including differences between healthy and disease-derived cells. Consequently, editing efficiencies may vary across loci despite standardized conditions.

Overall, this standardized and modular framework provides a robust platform for precise genome modification in primary human T cells, supporting both mechanistic investigation and translational gene editing applications.

### 13.1 Safety considerations

CRISPR/Cas9 -mediated cutting can result in undesired off-target editing (Tsai et al., 2015) and larger chromosomal changes at the target site (Tsuchida et al., 2023). To address some of these safety concerns, we previously studied genomic, transcriptomic and proteomic alterations in the edited T cells from the platform using GUIDE-seq, single-cell RNA sequencing, long-read genome sequencing and mass spectrometry, respectively (Mamia et al., 2025; Tsai et al., 2015). While these methods showed no safety concerns for the selected reagents, we advise diversifying safety profiling beyond these methods, including the use of *in silico* prediction tools, such as CHOPCHOP (Labun et al., 2019). Alternative methods to assess gRNA safety exist, such as CIRCLE-seq, SITE-seq, CHANGE-seq which all have their own advantages and limitations (Cromer et al., 2023). Alternatively, we advise performing timepoint experiments with extended T cell culture, along with *in vivo* xenotransplant studies, which can further complement assessing the safety of the platform and help evaluate the long-term T cell survival and risk of malignant transformation.

Alternative precision gene editing tools, such as base- (McAuley et al., 2023) and prime editing (Daliri et al., 2024), have emerged as safer alternatives to CRISPR/Cas9 as they do not induce double-strand DNA breaks. However, identifying safe and efficient guides remains a limiting factor for both approaches, as large-scale screening is often required to identify an effective prime editing guide RNA (pegRNA), while base editing may offer limited guide options and carries a risk of bystander editing at nearby coding bases.

### 13.2 Clinical implications and future directions

This T cell editing platform provides a practical framework for developing autologous cell therapies for IEIs and other monogenic disorders. By enabling targeted correction of disease-causing variants in patient-derived T cells, the approach supports restoration of endogenous gene function while preserving cellular characteristics important for therapeutic application.

The detailed protocol described here translates this strategy into a reproducible and standardized workflow, including CRISPR reagent design, gene editing and T cell quality control. Standardization of these steps can improve reproducibility across laboratories, facilitate generation of robust preclinical data, and support future clinical translation of gene-edited cell products. Translation to clinical manufacturing will further necessitate validation under GMP-compatible processes. Future efforts may focus on improving HDR efficiency and expanding the approach to more complex or multiplexed edits. Together, these advances may help accelerate the development of personalized genome-editing therapies for a broad range of rare genetic diseases.

Institutional permissions: The study was conducted in accordance with the principles of the Helsinki Declaration and approved by the Helsinki University Central Hospital Ethics Committee, and the Regional Committee for Medical and Health Research Ethics South-East Norway. All participants have signed written informed consent.

## Data Availability

The original contributions presented in the study are included in the article/supplementary material, further inquiries can be directed to the corresponding authors.
